# Rehmannioside A Attenuates Inflammation *via* Inactivation of the p-p65 NF-κB and p-p38 MAPK Signaling in Abdominal Infection-Induced Acute Kidney Injury

**DOI:** 10.33549/physiolres.935676

**Published:** 2025-12-01

**Authors:** Siyu XU, Tao HAN, Ye JIANG, Rongming LIU, Zunpeng SUN, Weixing GE, Yuyu LU, Ling WU, Cui FENG

**Affiliations:** 1 Department of Critical Care Medicine, The Affiliated Jiangning Hospital of Nanjing Medical University, Nanjing, China

**Keywords:** Acute kidney injury, Inflammation, Rehmannioside A, Renal dysfunction, Sepsis

## Abstract

Septic acute kidney injury (AKI) is the most common type of acute kidney failure observed in hospitalized patients, with inflammation playing a central role in its pathogenesis. The study aimed to investigate the influence of Rehmannioside A (Re A), a natural ingredient from the traditional Chinese herb Rehmanniae radix, on renal dysfunction and inflammation in septic AKI. Peripheral venous blood specimens were obtained from septic patients with and without AKI for comparing their clinical profiles. A rat model of sepsis was established through cecal ligation and puncture (CLP) surgery, followed by intraperitoneal injections of Re A. Biochemical analysis was performed to measure concentrations of kidney function indicators including blood urea nitrogen (BUN) and serum creatinine (Scr). Structural changes in kidney samples were evaluated by hematoxylin-eosin staining. Colony forming units of bacteria were counted in blood and renal samples of rats. Enzyme-linked immunosorbent assay was performed to measure serum and renal levels of proinflammatory cytokines (TNFα and IL-1β). Protein levels of factors related to MAPK and NF-κB pathways were quantified by western blotting. AKI patients showed increases in sepsis-related organ failure assessment (SOFA) score, BUN, Scr, TNFα and IL-1β levels compared to non-AKI patients. Re A improved the survival rate of CLP model rats and reversed CLP-induced increase in BUN and Scr levels. Pathological changes including renal tubular swelling and luminal narrowing induced by CLP were effectively ameliorated by Re A. In addition, Re A reduced bacterial load and proinflammatory cytokine levels in both blood and renal samples. As to the mechanism, Re A inactivated the phosphorylated levels of p38 MAPK and p65 NF-κB rat kidney samples. Re A plays renal-protective and anti-inflammatory properties in the rat model of septic AKI by inhibiting the activation of p38 MAPK and p65 NF-κB signaling.

## Introduction

Sepsis is known as a systemic inflammatory response syndrome triggered by infection, primarily featured by an overproduction of inflammatory factors [1]. It can lead to severe impairment of multiple organs, especially the heart and kidneys [2,3]. Septic shock, the most common types of circulatory shock, is associated with a hospital mortality exceeding 40 % [3]. Notably, intra-abdominal infection is the primary cause of sepsis in intensive care unit patients [4]. The incidence rate of acute renal injury (AKI) in patients with severe sepsis can amount to 51 % [5]. Therefore, effective prevention and management of AKI are crucial for reducing sepsis-related mortality.

It was previously thought that renal hypotension and the resulting ischemia were main factors in septic AKI; however, recent studies using various animal models have demonstrated that while tubular cell damage and the expression of markers like KIM-1 are prevalent, inflammation and apoptosis also key contributors [6]. Proinflammatory cytokines (IL-6, IL-1β, and TNFα) and anti-inflammatory factors (IL-10 and TGFβ) are main inflammatory cytokines associated with septic AKI [7,8]. Nuclear transcription factor-κB (NF-κB) serves as a crucial regulator of inflammation-related genes [9,10]. Upon activation, NF-κB promotes the transcriptional production of inflammatory factors and initiates a cascade of inflammatory mediators, playing a significant role in the development and progression of sepsis [5,11]. Research has shown that mitogen-activated protein kinases (MAPK) pathway is involved in the activation of NF-κB, which is essential for the expression of pro-inflammatory genes (i.e., TNF-α and interleukins), cyclooxygenase-2 and inducible nitric oxide synthase [12].

Increasing studies indicate that natural components play a promising role in the treatment of AKI [13–15]. For example, Trigonelline, a pyridine alkaloid from fenugreek, has been demonstrated to ameliorate renal function, oxidative stress, and inflammation in experimental sepsis AKI mouse model through the activation of NAD+/SIRT1 pathway [13]. Cichoric acid alleviates lipopolysaccharide-induced renal apoptosis, inflammation, and oxidative stress in AKI model mice [14]. Rehmannioside A (Re A) is a natural compound extracted from the traditional Chinese herb Rehmanniae radix [16]. As shown by previous studies, Re A can repress apoptosis, inflammation, and oxidative stress in the hippocampus of rats with vascular dementia, thereby improving cognitive function of experimental animals [17]. In addition, Re A plays an anti-psoriatic role by repressing inflammatory response and abnormal proliferation of HaCaT cells [16]. Moreover, Re A ameliorates inflammation and neuronal apoptosis after spinal cord injury by inhibiting p38 MAPK and p65 NF-κB signaling [18]. These discoveries demonstrate the anti-inflammatory property of Re A in various diseases. Nevertheless, the role of Re A in sepsis and AKI is obscure.

The study aimed to investigate whether Re A can protect experimental rats from cecal ligation and puncture (CLP)-induced AKI by regulating p38 MAPK and p65 NF-κB pathways. This study may provide a novel natural component effective for the treatment of septic AKI.

## Materials and Methods

### Patients and sampling

This study included 60 sepsis patients in the Nanjing Jiangning Hospital according to the diagnostic criteria established by the International Sepsis Definition Conference [19]. Among them, 38 patients were diagnosed with AKI and the rest 22 patients were defined as non-AKI group during the hospitalization. The clinical and demographic features of patients are provided in Table 1. Written informed consent had been obtained from each patient. This study was approved by the Ethics Committee of Nanjing Jiangning Hospital (2024-03-112-K01, 2024-03-11). The exclusion criteria included the following: (a) age under 18 years; (b) death occurring within 72 h of hospital admission; (c) exposure to nephrotoxins within 4 weeks prior to admission; (d) presence of pre-existing AKI, immunodeficiency, viral myocarditis, coagulation abonormalities or severer hepatitis; and (e) lack of treatment adherence. Upon hospital admission, peripheral venous blood samples were promptly collected from the patients, with no treatment administered prior to sampling.

### Animals

Sprague Dawley rats (25 ± 2 g) were purchased from Vital River Animal Technology (Beijing, China). These rats were kept in a standard room with constant temperature at 22 ± 2 °C and 50 % humidity. All rats had free access to water and food during the whole experimental process. The experimental protocols were under approval of the Institutional Animal Care and Use Committees of Nanjing Medical University (IACUC-2406091, 2024.06.09).

### Rat models of septic AKI

Rats were randomly assigned into four groups: sham (n=8), Re A (n=8), CLP (n=18), and CLP + Re A (n=18). Induction of cecal ligation and puncture (CLP) was performed as previously described [20]. The rats were anesthetized with isoflurane before modeling. A midline incision approximately 2 cm below the diaphragm was performed to reveal the cecum. After that, a 5-0 silk suture was used to ligate the junction of the cecum and colon. The cecum was then punctured twice using a 21 G needle. Afterward, it was repositioned, and the abdominal cavity was closed. Subsequently, each rat received subcutaneous resuscitation with 5 ml/100 g of sterile normal saline immediately after the procedure. Rats in the sham group received similar surgery without ligation and puncture of the cecum. For drug administration, rats in Re A groups were intraperitoneally injected with 80 mg/kg Re A 2 h after modeling, while animals in another two groups received intraperitoneal injections with the same amount of normal saline. The dosage of Re A was identified according to a previous study [18]. Seventy-two hours after surgery, rats were sacrificed after anesthesia with 3 % pentobarbital podium. For each group, 8 rats were identified for subsequent experiments. All blood and kidney samples collected from rats were stored at −80 °C until use.

### Measurement of renal function indicators

Blood samples collected from the hearts of rats were centrifuged at 1,000× g for 15 min at 4 °C to obtain rat serum samples. Human peripheral venous blood samples were centrifuged at 3000 r/m for 10 min to isolate serum. An automatic biochemical analyzer (Roche, Basel, Switzerland) was used to measure blood urea nitrogen (BUN) and serum creatinine (Scr) levels in rat or human serum samples.

### Hematoxylin-eosin staining

The right kidneys were collected and cut transversely in the middle. Next, renal tissues were preserved in 10 % formalin, embedded in paraffin, and sectioned into 4 μm thickness. The sections were then deparaffinized in xylene for 30 min and hydrated in graded ethanol (100 %, 95 %, 80 %, and 70 %). After washing with phosphate buffered saline in triplicate, the sections were stained with hematoxylin and eosin for 3 min and finally mounted with neutral resin. An optical microscope (Olympus, Tokyo, Japan) was employed to observe the results of staining.

### Detection of bacterial load

After the harvest of the left kidney and the removement of surrounding fat and fascia, the remaining tissues were homogenized in ultrathurax tubes and mixed with brain-heart infusion broth (BHI) by vortexing. A solution was prepared by combining 900 μL of suspension with 100 μL of glycerol (99 %). For blood samples, 50 μL of sterile whole blood was mixed with 30 μL of BHI, and 20 μL of glycerol (50 %). To assess bacterial load, the renal or blood samples were diluted at 1:100 using BHI. A volume of 25 μL from the suspension was then inoculated onto sheep blood agar and incubated for 24 h at 37 °C in aerobic conditions. The colony-forming units (CFUs) were counted and reported as log CFU per mL of undiluted blood or CFU per gram of undiluted tissue homogenate.

### Enzyme-linked immunosorbent assay (ELISA)

The rat blood samples were harvested in anticoagulative tube with ethylenediaminetetraacetic acid, followed by centrifugation at 3000×g for 10 min. Levels of TNFα and IL-1β in serum samples were measured by rat TNFα ELISA kit (ab236712, Abcam, UK) and rat IL-1β ELISA kit (ab255730, Abcam), while those in renal samples were assessed using rat TNFα ELISA kit (ab100785, Abcam) and rat IL-1β ELISA kit (ab255730, Abcam). For detection of TNFα and IL-1β levels in human serum samples, human TNFα ELISA kit (ab181421, Abcam) and human IL-1β ELISA kit (ab242234, Abcam) were used, following the manufacturer’s recommendations. The absorbance values were measured at 450 nm.

### Western blotting

Total protein in kidney tissues was extracted by adding tissue lysis buffer (Roche, Basel, Switzerland). A bicinchoninic acid kit (Thermo Fisher Scientific, Waltham, MA, USA) was used to detect protein concentration. The protein contents were subjected to electrophoresis and then transferred to a polyvinylidene fluoride at 4 °C. Afterwards, the membrane was blocked with 5 % bovine serum albumin before overnight incubation with primary antibodies at 4 °C. After washing with Tris buffered saline with Tween-20, the membrane was incubated with secondary antibodies at 37 °C for 1 h. Enhanced chemiluminescence reagent (Solarbio, Beijing, China) was used to illuminate the bands, and Quantity One software was used to analyze relative protein expression with normalization to GAPDH level. Primary antibodies used in the study include anti-p38 MAPK (ab308333, 1:1000), anti-phosphorylated (p)-p38 (ab60999, 1:500), anti-p65 NF-κB (ab16502, 1:500), anti-p-p65 (ab76302, 1:1000), and anti-GAPDH (ab9485, 1:2500).

### Statistical analysis

All data are shown as the mean ± standard deviation. Using GraphPad Prism 8, differences among four groups were compared using one-way analysis of variance and Tukey’s *post hoc* test. The threshold for statistical significance was p<0.05.

## Results

### Patients with AKI display abnormal increases in renal function indicators and proinflammatory cytokines

Blood samples were collected from sepsis patients diagnosed with AKI (AKI group, n=38) and without AKI (non-AKI group, n=22) to analyze their clinical characteristics. As shown in Table 1, no significant differences were found in age, gender, body mass index (BMI), or white blood cell count (WBC) between the AKI group and non-AKI group (p>0.05). Notably, AKI patients showed an increase in sepsis-related organ failure assessment (SOFA) score compared to non-AKI patients (11.14±3.68 vs 7.85±2.63) (Table 1). Moreover, serum levels of renal function indicators (Scr and BUN) were significantly increased in AKI patients relative to non-AKI patients (Scr: 158.64±32.58 μM vs 95.58±22.42 μM; BUN: 1.88±0.53 mmol vs 0.65±0.12 mmol) (Table 1). Serum concentrations of the proinflammatory cytokines (TNFα and IL-1β) were markedly elevated in AKI patients compared to non-AKI patients (TNFα: 82.75±20.12 pg/ml vs 52.48±12.26 pg/ml; IL-1β: 23.15±5.69 pg/ml vs 4.48±0.53 pg/ml) (Table 1). These findings indicate that AKI is clinically associated with more severe sepsis, renal dysfunction, and inflammatory response, thereby providing a rationale for subsequent animal experiments.

### Rehmannioside A (Re A) improves the survival rate of CLP model rats

**Fig. 1:**
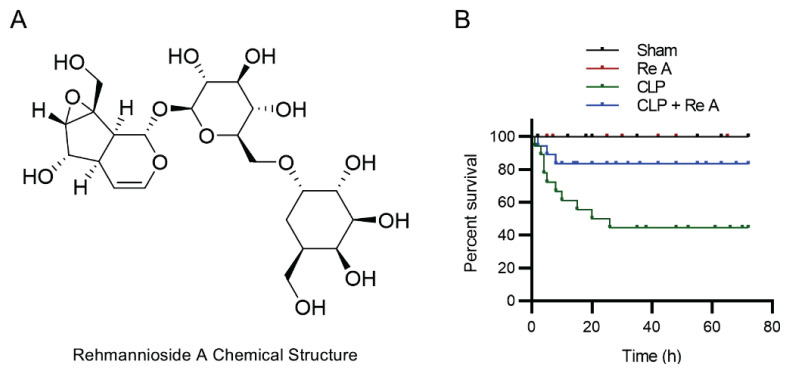
Chemical structure of Rehmannioside A (Re A) and its effect on the survival rate in sham rats or CLP-induced septic rats. **A**) Chemical structure of Rehmannioside A (C21H32O15). **B**) The survival rate of rats in sham group, Re A group, CLP group, and CLP + Re A group.

A rat model of CLP was established to investigate the role of Re A (C_21_H_32_O_15_, Fig. 1A) in septic AKI. The survival of rats after modeling and Re A administration was recorded and shown in Figure 1B. Re A treatment did not cause death of sham-operated rats, with the survival rate of 100 %. CLP treatment significantly reduced the survival rate (green line). However, in response to Re A administration, the survival of CLP rats was significantly improved (blue line) (Fig. 1B).

### Re A improves renal dysfunction of CLP rats

**Fig. 2:**
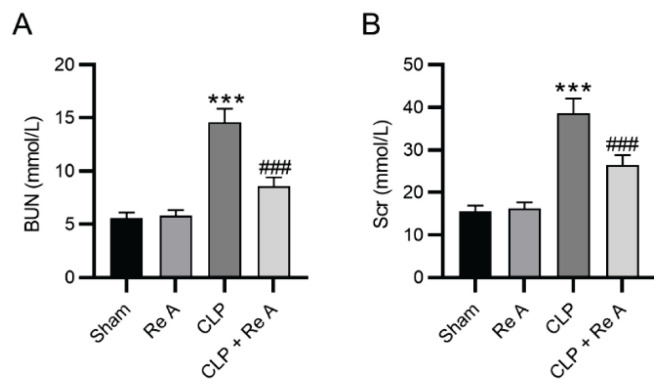
Effects of Re A on renal function indicators. **A–B**) A biochemical analyzer was used to measure contents of renal function indicators of blood urea nitrogen (BUN) and serum creatinine (Scr) in rat blood samples. ***p<0.001 versus sham group, ^###^p<0.001 versus CLP group.

To verify whether Re A exerts a protective effect against CLP-induced kidney injury, two renal function indicators in blood samples were subjected to biochemical analysis. Blood urea nitrogen (BUN) and serum creatinine (Scr) levels were prominently increased in response to CLP induction compared with their levels in the sham group (Fig. 2A,B, p<0.001). Administration of Re A exerted no significant effects on BUN and Scr levels in sham-operated rats; however, Re A treatment significantly (p<0.001) alleviated CLP-induced increase in BUN and Scr levels in rats (Fig. 2A–2B). The finding suggests that Re A improves kidney dysfunction in CLP rats.

### Re A restores pathological changes in renal tissues of CLP rats

**Fig. 3:**
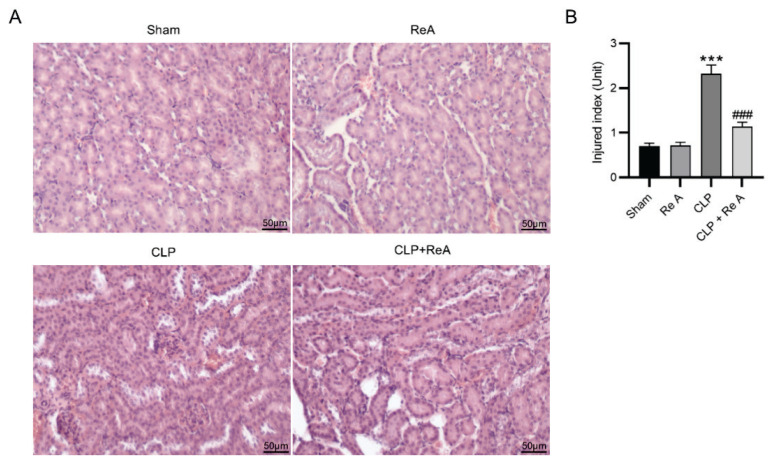
Re A ameliorates pathological changes in renal tissues of CLP rats. **A**) Hematoxylin-eosin staining was performed to measure pathological changes in rat renal tissues. **B**) Renal injury index in sham, Re A, CLP, and CLP + Re A groups was scored. ***p<0.001 versus sham group, ^###^p<0.001 versus CLP group.

To further explore the influence of Re A on septic AKI, hematoxylin-eosin staining was performed to observe pathological changes caused by CLP and Re A. As displayed in Figure 3A, normal tubular morphologies and obvious lumens were discovered in the sham group and the Re A group. CLP surgery induced obvious renal tubular swelling and luminal narrowing, and these changes in model rats were effectively ameliorated in the context of Re A treatment (Fig. 3A). Renal tubular damage in each group was scored in Figure 3B. Compared with the injury index in the sham group, that in CLP group was markedly elevated (2.32±0.2 vs 0.7±0.07, p<0.001). Re A reduced the high injury score induced by CLP surgery (Re A + CLP group vs CLP group: 1.14±0.1 vs 2.32±0.2, p<0.001) while exerting no changes to the injury score in Re A group relative that in the sham group (0.72±0.07 vs 0.7±0.07) (Fig. 3B). The results indicated that CLP-induced kidney injury was effectively mitigated by Re A treatment.

### Re A reduces bacterial load and proinflammatory cytokine levels in septic AKI

**Fig. 4:**
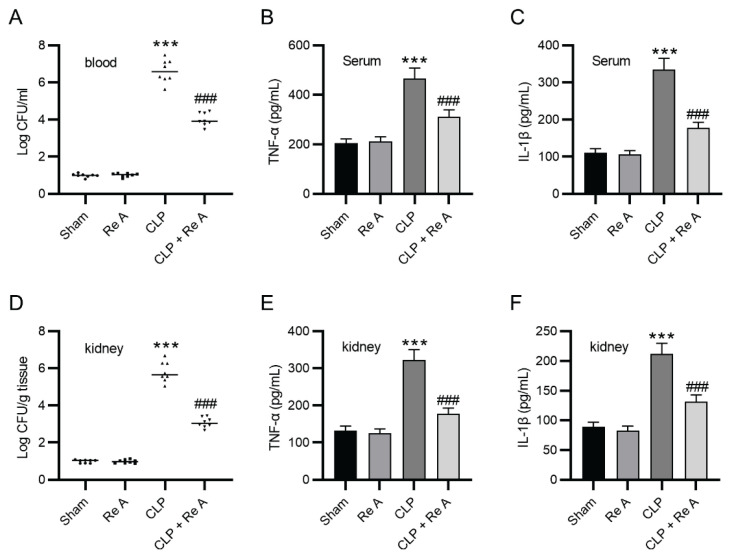
Re A alleviates bacterial proliferation and decreases proinflammatory cytokine levels in septic AKI. **A**) Colony forming units (CFUs) of bacteria in rat blood samples were counted. **B–C**) ELISA was performed to measure serum levels of proinflammatory factors (TNFα and IL-1β) in each group. **D**) CFUs of bacteria in rat kidney samples were determined. **E–F**) Concentrations of inflammatory cytokines in rat renal tissues were assessed using ELISA. ***p<0.001 versus sham group, ^###^p<0.001 versus CLP group.

Increasing evidence shows that systematic and kidney bacterial load is a critical trigger for inflammation and renal damage during sepsis [20,21]. To explore whether Re A exerts its renal protective role in septic AKI via anti-bacterial and anti-inflammatory properties, colony forming units (CFUs) of bacteria and proinflammatory cytokine levels in blood samples and renal tissues were assessed. Figure 4A revealed that bacterial load in rat blood samples of CLP rats was markedly increased compared with that in sham-operated rats (p<0.001), and the high bacterial load in CLP rats was significantly reduced in response to Re A administration (p<0.001). Serum levels of proinflammatory cytokines (TNFα and IL-1β) were largely accumulated in the CLP group relative to those in the sham group, and the alterations induced by CLP were counteracted by Re A treatment (Fig. 4B,C, p<0.001). Consistently, Re A administration effectively diminished CLP-induced high bacterial load, TNFα level, and IL-1β level in rat kidney samples (Figure 4D–F). Overall, Re A plays anti-bacterial and anti-inflammatory activities in septic AKI.

### Re A inhibits the activation of MAPK and NF-κB signaling in rats with septic AKI

**Fig. 5:**
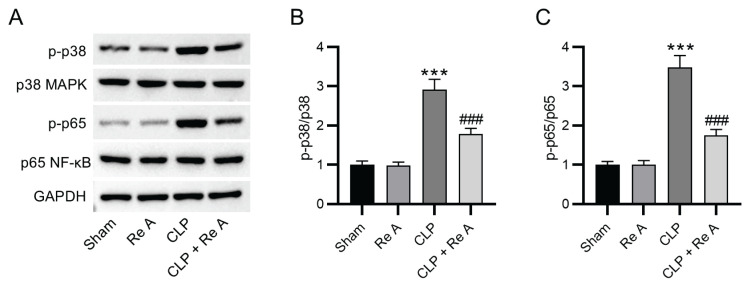
Re A suppresses the activation of MAPK and NF-κB signaling in rats subjected to septic AKI. **A**) Western blotting was performed to measure protein levels of p38 MAPK, p65 NF-κB, and their phosphorylated (p) levels in each group. **B–C.** Quantification of p-p38/p38 and p-p65/p65 ratios in each group. ***p<0.001 versus sham group, ^###^p<0.001 versus CLP group.

To investigate the mechanism underlying the anti-inflammatory effect of Re A in septic AKI, protein levels of key factors involved in the MAPK and NF-κB signaling pathways were measured by western blotting. The ratio of phosphorylated (p)-p38 MAPK level to p38 MAPK level as well as the ratio of p-p65 NF-κB level to NF-κB level were noticeably elevated in renal tissues of CLP rats, and the trend was reversed by Re A treatment (Fig. 5A–5C, p<0.001). The finding indicates that Re A suppressed CLP-induced activation of MAPK and NF-κB pathways in rat renal tissues.

## Discussion

Systemic inflammation and organ dysfunction are main features of sepsis [22,23]. One of the most commonly affected organs is the kidney, leading to sepsis-associated AKI, which significantly contributes to the morbidity and mortality of sepsis [6]. In this study, peripheral venous blood samples were collected from sepsis patients with and without AKI to compare their clinical characteristics. Patients who developed AKI exhibited significantly higher sepsis-related organ failure assessment (SOFA) score compared to those without AKI, suggesting more pronounced organ injury. The SOFA score, also referred to as the sequential organ failure assessment score, comprises six distinct components corresponding to the respiratory, renal, hepatic, cardiovascular, coagulation, and neurological systems. Elevated SOFA scores reflect greater severity of organ dysfunction and are associated with worse clinical outcomes [24,25]. In addition, levels of kidney function indicators (BUN and Scr) and proinflammatory factors (TNFα and IL-1β) were found to be increased in sepsis patients with AKI compared to those without AKI. These results demonstrate that septic AKI is closely associated with more severe systemic organ injury, impaired renal dysfunction, and excessive inflammatory response.

Moreover, a rat model of CLP was established to mimic abdominal infection-induced septic AKI. We observed significantly elevated levels of BUN and Scr in serum samples as well as renal tubular swelling and luminal narrowing in CLP-treated rats. These structural changes in kidneys are consistent with previous studies focused on septic AKI [26–29]. Specifically, Show *et al.* found evident edema, enhanced inflammatory infiltration, degenerated epithelial cells, narrowed renal tubules, and enlarged glomerular in rat kidneys [26]. Using another animal model of septic AKI, Ren *et al.* reported that lipopolysaccharide induced tubular dilation, swelling, and disappearance of brush border in mice [27]. These consistent pathological changes suggest that the animal model was successfully established. Furthermore, bacterial lipopolysaccharide serves as a trigger for initiation of the systemic inflammatory reaction in sepsis. An uncontrolled inflammatory response may contribute to organ failure and adverse outcomes in septic patients [20,30]. In this study, the bacterial load in blood and renal samples as well as serum levels of TNFα and IL-1β were elevated in rats, indicating the bacterial propagation and abnormal inflammatory response induced by septic AKI.

According to previous studies, two ingredients of Radix rehmanniae have been reported to ameliorate septic diseases, which are catalpol and 2,5-Dihydroxy-acetophenone [31,32]. Catalpol was demonstrated to play a neuroprotective role in septic-associated encepha-lopathy by reversing neuroinflammation through inhibition of the NF-κB pathway [31]. 2,5-Dihydro-xyacetophenone, an extract sourced from Radix rehmanniae praeparata, exerts an anti-inflammatory role in CLP-induced AKI by inhibiting ERK and NF-κB signaling [32]. The current study first validated that another component in Radix rehmanniae, namely Re A, plays a renal protective role in the alleviation of septic AKI by blocking the p38 MAPK and p65 NF-κB pathways.

The two signaling and their downstream/upstream factors have been widely verified to be involved in the progression of septic AKI [33–36]. Sun *et al.* reported that inhibition of NF-κB/STAT3/MAPK pathway leads to the reduction of bacterial load and inflammatory mediators (TNFα, IL-1β, and IL-6) [37]. Inhibition of WIP1-mediated p38 MAPK signaling ameliorates pyroptosis in lipopolysaccharide-triggered septic AKI [38]. The mechanism elucidated in the present study is consistent with those described in previous articles.

In conclusion, Re A ameliorates abdominal infection-induced AKI through inhibition of inflammatory response via inactivation of the p38 MAPK and p65 NF-κB signaling. The study first revealed the significance of Re A in septic diseases and its renal protective effects. However, the study mainly primarily focuses on inflammatory response and lacks investigation of other biological processes such as oxidative stress, apoptosis, and pyroptosis, which is a limitation of this study. Future studies should further explore the renal protective effects of Re A, which may lead to its development as a promising therapeutic agent for septic AKI.

## Figures and Tables

**Table 1:** Clinical characteristics of patients with sepsis.

Characteristics	Non-AKI (n=22)	AKI (n=38)	P value
Age (years)	52.15±15.23	50.47±14.98	0.6789
Gender (male/female)	17/5	31/7	0.6878
BMI (kg/m^2^)	22.35±2.41	22.74±2.44	0.5513
SOFA score	7.85±2.63	11.14±3.68	0.0005
WBC (×109 /L)	14.02±7.25	15.65±8.53	0.4550
Scr (μM)	95.58±22.42	158.64±32.58	<0.0001
BUN (mmol)	0.65±0.12	1.88±0.53	<0.0001
TNF-α (pg/ml)	52.48±12.26	82.75±20.12	<0.0001
IL-1β (pg/ml)	4.48±0.53	23.15±5.69	<0.0001

BMI: body mass index, SOFA: Sepsis-related Organ Failure Assessment, WBC: white blood cell, Scr: serum creatinine, BUN: blood urea nitrogen, TNF-α: tumor necrosis factor-α, IL-1β: interleukin-1β.
